# The role of microRNA-133b and its target gene FSCN1 in gastric cancer

**DOI:** 10.1186/s13046-014-0099-0

**Published:** 2014-11-30

**Authors:** Lihua Guo, Hua Bai, Dongling Zou, Tao Hong, Jie Liu, Jiaqiang Huang, Pengfei He, Qi Zhou, Jinsheng He

**Affiliations:** School of Computer and Information Technology, Shangyuan Residence, Haidian District Beijing, 100044 China; College of Life Sciences and Bioengineering, Beijing Jiaotong University, Shangyuan Residence, Haidian District Beijing, 100044 China; Department of Ophthalmology, General Hospital of Bei Jing Command of PLA, #5 Nanmencang, DongCheng District Beijing, 100700 China; Department of Gynecologic Oncology, Chongqing Cancer Institute, Chongqing, 400030 China; National Institutes for Food and Drug Control, No.2 Tiantan Xi Li, Beijing, 100050 China

**Keywords:** Gastric cancer, miR-133b, FSCN1, Tumor suppressor

## Abstract

**Background:**

Increasing evidences have documented that microRNAs (miRNAs) act as oncogenes or tumor suppressors in gastric cancer (GC). In this study, we aimed to investigate the expression of miR-133b in a large number of GC samples and elucidate its role in GC carcinogenesis and the detailed mechanism.

**Methods:**

We used Taqman probe stem-loop real-time PCR to accurately measure the levels of miR-133b in 100 pairs of gastric cancer tissues and the adjacent non-neoplastic tissues. miR-133b mimics were overexpressed in GC cell lines, miR-133b inhibitors were also introduced in GES cells to investigate its role on regulating cell proliferation, cell migration and cell invasion. The target of miR-133b was identified by luciferase reporter assay and western blot. Fascin actin-bundling protein 1 (FSCN1) siRNA was used to achieve the knockdown of FSCN1 in GC cells and to investigate its role on modulating GC cell proliferation and invasion.

**Results:**

miR-133b was significantly down-regulated in GC cell lines and in GC tissues compared with adjacent normal tissues. Moreover, lower-level of miR-133b was also associated with venous invasion and a more aggressive tumor phenotype. Re-introduction of miR-133b in GC cells can inhibit cell proliferation, cell migration and invasion. In contrary, knockdown of miR-133b in GES cells can promote cell proliferation and invasion. Further investigation indicated that miR-133b targeted FSCN1 in GC cells and knockdown of FSCN1 can also inhibit GC cell growth and invasion.

**Conclusion:**

Our findings demonstrated that miR-133b was significantly down-regulated in GC tissues and exerted its tumor suppressor role in GC cells. The investigation of the detailed mechanism showed that miR-133b directly targeted FSCN1 which functioned as an oncogenic gene in GC cells. These results suggested that miR-133b can be developed as a new diagnostic marker or therapeutic target for GC.

## Background

Gastric cancer is the second most common cause of cancer-related death in the world. Diverse treatment strategies including surgery, chemotherapy and radiotherapy can relieve the pain and lessen the possibility of systemic metastasis. However, the overall therapeutic activity for advanced disease remains poor [1]. To discover and identify new biomarkers for earlier stages of GC or specific biomarkers for different individuals is urgently required for early detection of cancer and individualized therapies. Recently, the roles of microRNAs (miRNAs) as potential biomarkers and therapy targets have been widely investigated in many kinds of cancers.

MiRNAs are endogenous small non-coding RNA molecules, which function in transcriptional and post-transcriptional regulation of gene expression. Accumulating evidence demonstrate that miRNAs play important roles in a variety of biological processes and the deregulation of miRNAs is involved in many diseases [2,3]. Numerous studies have documented that miRNAs acted as oncogenes or tumor suppressors in diverse cancers, such as lung, breast, hepatic, pancreatic cancer and gastric cancer [4-11]. Currently, the aberrant expression of many miRNAs has been observed in GC. For example, miR-21, miR-124, miR-125b, miR-221-222 cluster, miR-106b-25 cluster have been shown to contribute to gastric carcinogenesis by changing the cell cycle, cell apoptosis, cell migration and invasion through targeting the relative genes [12,13]. In addition, a lot of miRNAs, especially circulating miRNAs, have been shown to be associated with tumor stages or patient survival, and might be developed as potential biomarkers for GC diagnosis [14]. Therefore, exploring the aberrant expression pattern of miRNAs and the roles of miRNAs in GC will be benefit to understand the mechanism of GC carcinogenesis and develop new methods for GC diagnosis and therapy.

miR-133b, which is initially considered to be a muscle-specific miRNA [15,16], has been reported to be deregulated in many kinds of cancer [17-22]. The down-regulation of miR-133b in gastric cancer has also been reported by several groups [23,24]. Recently, miR-133b is found to negatively regulate FGFR1 in gastric cancer and might act as a tumor suppressor in GC [25]. However, in these studies, the expression of miR-133b was detected only in a small number of GC tissues or just in the GC cell lines. The expression of miR-133b in a large number of clinical samples was not determined. miR-133b has been reported to directly target oncogenic Fascin actin-bundling protein 1 (FSCN1) in esophageal squamous cell carcinoma [26]. In GC, FSCN1 also might act as an oncogene and the high level of FSCN1 was significantly correlated with shorter survival time and several aggressive pathological factors [27]. In addition, FSCN1 mRNA was upregulated in accordance with miR-133b down-regulation in GC patients [24]. However, the tumor suppressor roles of miR-133b in GC and the detailed mechanism are largely unknown.

In this study, we used Taqman probe stem-loop real-time PCR to accurately measure the levels of miR-133b in 100 pairs of gastric cancer tissues and the adjacent non-neoplastic tissues. We found that miR-133b was significantly down-regulated in GC tissues and the lower level of miR-133b in GC was significantly associated with a more aggressive tumor phenotype. Re-introduction of miR-133b to the GC cells could inhibit the cell proliferation, migration and invasion. The mechanism study indicated that miR-133b directly targeted FSCN1 which functioned as an oncogenic gene in GC cells. These results suggested that miR-133b functioned its tumor suppressor’s role in GC possibly through down-regulating FSCN1.

## Methods

### Patients and specimens

The human clinical samples were collected from surgical specimens from 100 patients with gastric cancer at The Military General Hospital of Beijing PLA. The study was approved by the ethical board of the hospital and the ethical board of Beijing Jiaotong University. The corresponding adjacent non-neoplastic tissues from the macroscopic tumor margin were isolated at the same time and used as controls. Tumor and non-cancerous tissues were confirmed histologically by haematoxylin and eosin (H&E) staining. All samples were immediately snapped frozen in liquid nitrogen and stored at −80°C until RNA extraction.

### Cell culture and transfections

The human gastric cancer cell lines, including HGC-27, MGC-803, MKN-25 and SGC-7901 and normal human gastric epithelium cells (GES) were propagated in DMEM (Invitrogen) supplemented with 10% FCS at 37°C in 5% CO_2_ cell culture incubator. 293 T cells were cultured in DMEM medium supplemented with 10% FCS. miR-133b mimics, scramble control mimic, FSCN1 siRNA and siRNA control were obtained from Dharmacon (Austin, TX, USA) and transfected with DharmFECT1 (Dharmacon, Austin, TX, USA) in HGC-27 and MGC-803 cells at a final concentration of 50 nM.

### RNA extraction, cDNA synthesis, and real-time PCR assays

Total RNA was extracted from tissues and cells using Trizol reagent (Invitrogen, CA, USA) according to the manufacturer’s instructions. cDNA was synthesized by M-MLV reverse transcriptase (Invitrogen) from 2 μg of total RNA. A stem-loop RT primer was used for the reverse transcription of miR-133b. Quantitative RT-PCR was performed in a Bio-Rad CFX96 real-time PCR System (Bio-Rad, CA, USA) using TaqMan probes (Applied Biosystems, Foster City, CA, USA) according to the manufacturer’ s instructions. The PCR conditions were as follows: 95°C for 30 s, followed by 40 cycles of 95°C for 5 s and 60°C for 34 s. The data were normalized using the endogenous U6 snRNA. The 2-ΔΔCT method was used in the analysis of PCR data. Primer sequences are presented in Table 1.

**Table 1:** **Sequence of primers used in qRT-PCR and constructs**

**Primer**	**Sequence (5′ → 3′)**
miR-133b-RT	GTCGTATCCAGTGCAGGGTCCGAGGTATTCGCACTGGATACGACTAGCAGG
miR-133b-forward	CTGGAGTTTGGTCCCCTTCAAC
miR-133b-reverse	GTGCAGGGTCCGAGGT
miR-133b-probe	FAM-ATACGACTAGCAGGTTGA-MGB
U6-RT	AAAATATGGAACGCTTCACGAATTTG
U6-forward	CTCGCTTCGGCAGCACATATACT
U6-reverse	ACGCTTCACGAATTTGCGTGTC
U6-probe	FAM-CCATGCTAATCTTCTCTGTA-MGB
FSCN1-UTR-F	TGGGCTAGGACTGACCCTTGT
FSCN1-UTR-R	GGGAGCACCAATCACAGCA
FSCN1-UTR-Mut Sense	GAAAATCGCTCATTCAGTATTT
FSCN1-UTR-Mut Anti	TACTGAATGAGCGATTTTCTGCTT

### Cell proliferation assay and colony formation assay

To measure the effect of miRNA mimics or FSCN1 siRNA on cell proliferation, cells were incubated in 10% CCK-8 (DOJINDO) diluted in normal culture media at 37°C until visual color conversion appears. Proliferation rates were determined at day 1, 2, 3, 4 post-transfection, and quantification was done on a microtiter plate reader (Spectra Rainbow, Tecan) according to the manufacturer's protocol. Meanwhile, the mimic-transfected cells were trypsinized and replated at 200 cells per well in 6-well plates, cultured for 7 days, then fixed with methanol and stained with 0.1% crystal violet in 20% methanol for 15 min.

### Cell migration and invasion assays

A wound-healing assay was performed to assess the cell migration ability. An artificial wound was created on a confluent cell monolayer 24 hours after transfection. Images were taken at 0, 24, 48 hours and percentage of open wound was calculated.

HGC-27 and MGC-803 cells were seeded onto a Matrigel-coated membrane matrix (BD Bioscience) present in the insert of a 24 well culture plate. FBS was added to the lower chamber as a chemoattractant. After 24 hours, invasive cells located on the lower surface of chamber were stained with the 0.1% crystal violet (Sigma) and counted.

### Constructs and Luciferase assay

The reverse complementary sequence of miR-133b was inserted into pMIR-reporter (Promega, WI, USA) to generate a reporter system (pMIR-133b) to detect mature miRNA expression in 293 T cells. The 3′ UTR of the human FSCN1 was PCR amplified and cloned into pMIR-reporter downstream of the firefly luciferase gene to generate the corresponding reporters. Mutations at the miRNA binding site in these mRNA sequences were created using bridging PCR. For miRNA targets analysis, the 293 T cells were co-transfected with 0.4 μg of the reporter construct, 0.02 μg of pRL-TK vector, and 5 pmol of miRNA mimic or scramble controls. Cells were harvested 48 h post-transfection and assayed with Dual Luciferase Assay (Promega, WI, USA) according to manufacturer’s instructions. All transfection assays were carried out in triplicates.

### Western blotting

At the indicated times, MGC-803 cells and HGC-27 cells were collected and the whole-cell lysate was extracted using lysis buffer (0.05 M Tris, pH =7.5, 0.15 M NaCl, 2% NP-40) containing 200 lm Na3VO4, 200 mM NaF, 0.5 M EDTA, proteinase inhibitors, for 30 min on ice. The whole-cell lysate was quantified by BCA method according to manufacturer’s instructions. Proteins were separated by SDS-PAGE and then transferred to the NC membrane for the subsequent immunoblot analysis. The following antibodies were used for Western blot: GAPDH (10494-1-AP, Proteintech), FSCN1 (14384-1-AP, Proteintech).

### Statistics

The comparison of miR-133b expression between gastric cancer tissue and adjacent non cancer tissue was evaluated by Independent Samples T test (two-tailed). Correlation of miR-133b expression with patients’ clinicopathological variables was evaluated by independent sample T-test (two-tailed). P ≤0.05 was considered statistically significant.

## Results

### miR-133b is down-regulated in GC

**Figure 1:**
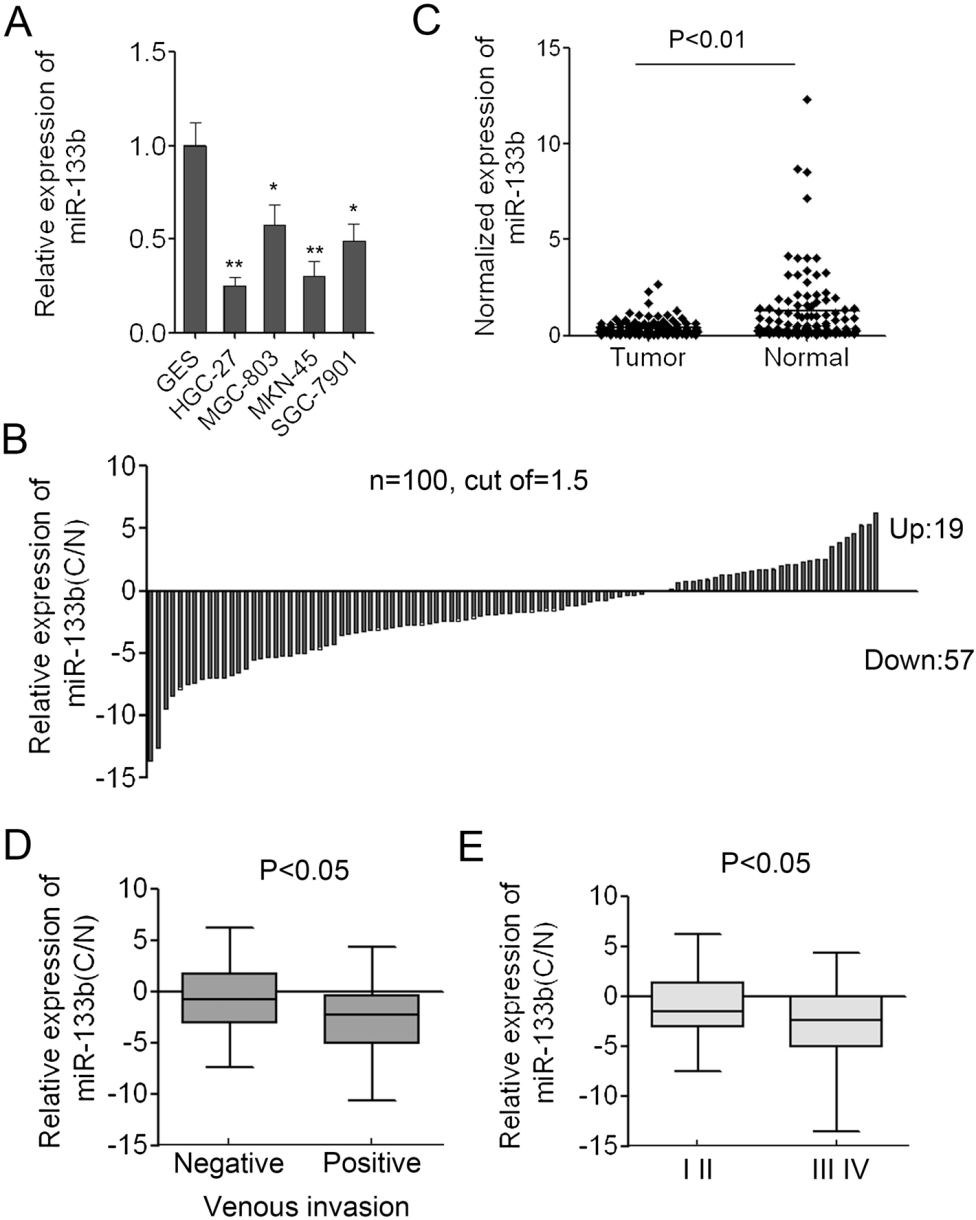
**miR-133b is down-regulated in GC cells and in GC tissues. (A)** The expression level of miR-133b in the four GC cell lines (HGC-27, MGC-803, SGC-7901 and MKN-45) and a human gastric epithelium cell line (GES). Data are shown as mean + s.d. (n = 3); * indicates P-value <0.05; ** indicates P-value <0.01. **(B)** The relative expression of miR-133b in 100 pairs of GC tissues (C) and adjacent non-neoplastic tissues (N). **(C)** Normalized expression of miR-133b in 100 pairs of GC and adjacent normal tissues. **(D)** The correlation of miR-133b expression with venous invasion. **(E)** The correlation of miR-133b expression with tumor stages.

To accurately analyze the expression of miR-133b in GC, q-PCR using Taqman probes was conducted to measure the levels of miR-133b. We firstly examined the expression of mature miR-133b in four human GC cell lines (HGC-27, MGC-803, SGC-7901 and MKN-45) and a human gastric epithelium cell line (GES). The expression level of miR-133b in the four GC cell lines was significant lower than that in GES and the expression in MKN-45 cells was the lowest (Figure 1A). Subsequently, we measured the levels of miR-133b in 100 pairs of gastric cancer tissues (C) and the adjacent non-neoplastic tissues (N). The results of PCR showed that 57/100 (57%) of cases had reduced levels of miR-133b in GC tissues compared with the corresponding non-neoplastic tissues when the cutoff was set up as 1.5 (Figure 1B). There were 19/100 (19%) of cases had increased levels of miR-133b in gastric cancer tissues compared with the adjacent normal tissues, 24/100 (24%) of cases in whom the expression of miR-133b in gastric cancer tissues was unchanged when the cutoff was set up as 1.5. The results also showed that the average expression of miR-133b in gastric cancer samples was significantly lower than that in the adjacent non-neoplastic tissues (p < 0.01) (Figure 1C). Collectively, the data indicated that miR-133b was significantly attenuated in tumor tissues compared with adjacent normal tissues and might act as a tumor suppressor in GC.

### Low-level expression of miR-133b is associated with aggressive phenotypes of GC

To further investigate the correlation between the expression of miR-133b and the clinicopathological characteristics, the relative expression of miR-133b in 100 pairs of gastric cancer tissues and adjacent normal tissues were statistically analyzed. The clinicopathological features of gastric cancer patients were summarized in Table 2. Correlation analysis showed that low-level of miR-133b in gastric cancer was associated with venous invasion (*p* < 0.05, Figure 1D) and a more aggressive tumor phenotype (*p* < 0.05, stage I/II vs. III/IV) (Figure 1E). However, no significant associations with age, gender, position, or borrmann typing were observed. The correlation of lower levels of miR-133b with the more aggressive phenotype of gastric cancer strongly indicated that miR-133b played important roles in gastric carcinogenesis.

**Table 2:** **Clinicopathological features of gastric cancer patients**

**Variables**	**Patients, n**	
	**Total**	**Lower miR-133b**
	**(n =100)**	**(n = 57)**
Histological type		
Well differentiated	23	9
Poorly differentiated	77	48
Gender		
male	73	41
female	27	16
Age		
≥60	58	30
<60	42	27
Venous invasion		
Negative	61	27
Positive	39	30
Nerve invasion		
Negative	59	32
Positive	41	25
Position		
Cardia	40	22
Gastric body	27	15
Gastric antrum	33	20
Borrmann typing		
I	10	7
II	74	40
III	16	10
pT stage		
T1T2	21	12
1T3T4	79	45
pN stage		
N0	23	15
N1N2N3	77	42
pM stage		
M0	80	40
M1	20	17
pTNM stage		
I + II	25	10
III + IV	75	47

### Re-introduction of miR-133b in GC cells can inhibit cell growth and colony formation

**Figure 2:**
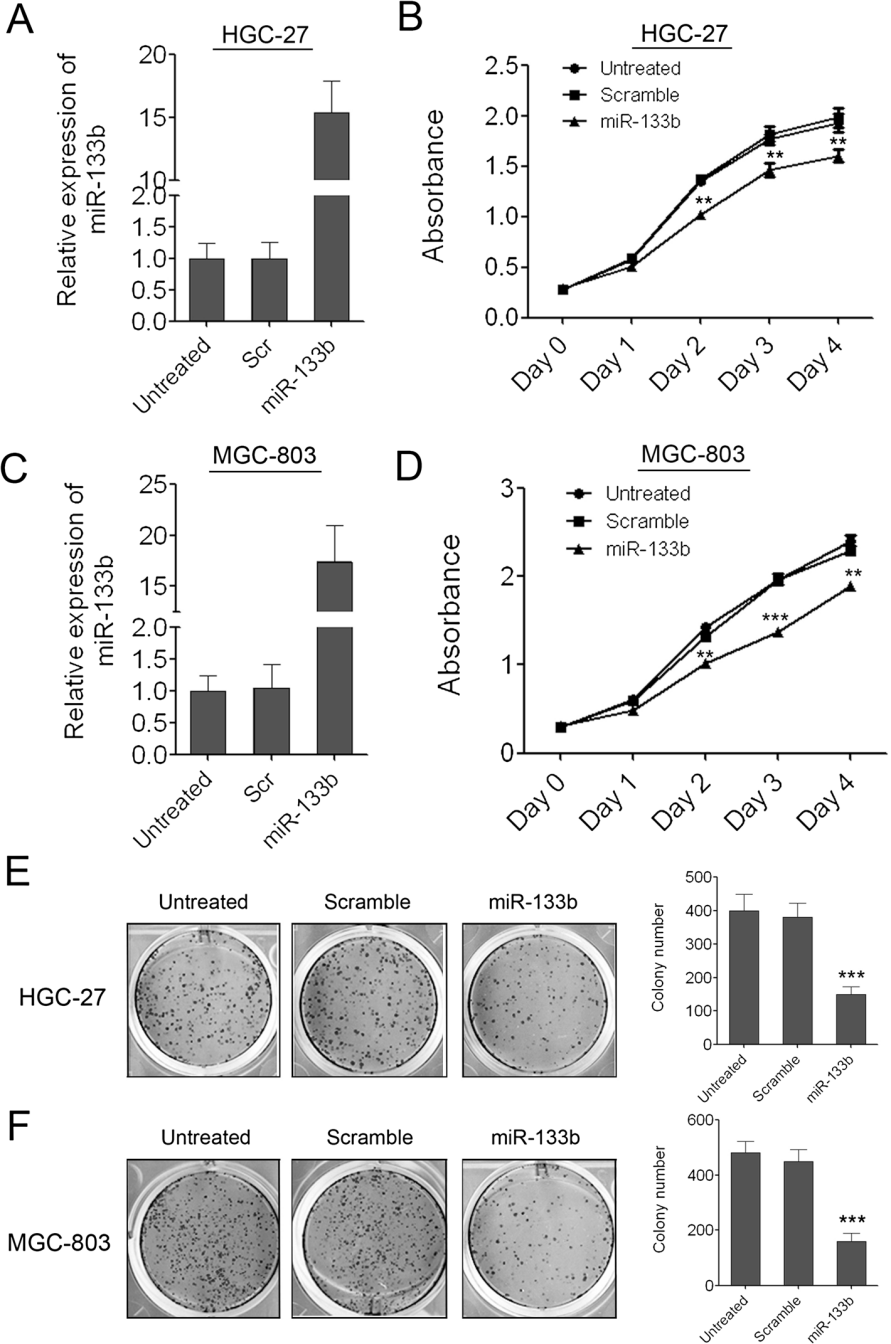
**Enforced expression of miR-133b suppresses cell proliferation and colony formation ability of GC cells. (A)** Overexpression of miR-133b in HGC-27 cells was confirmed by qRT-PCR. **(B)** The cell growth of HGC-27 cells at day 0, 1, 2, 3, 4 post transfection which was detected by CCK-8 assay. Data are shown as mean ± s.d. (n = 3); ** indicates P-value <0.01. **(C)** Overexpression of miR-133b in MGC-803 cells was confirmed by qRT-PCR. **(D)** The cell growth of MGC-803 cells at day 0, 1, 2, 3, 4 post transfection which was detected by CCK-8 assay. Data are shown as mean ± s.d. (n = 3); ** indicates P-value <0.01; *** indicates P-value <0.001. **(E)** The colony number of HGC-27 cells per well in 6-well plates cultured for 7 days. **(F)** The colony number of MGC-803 cells per well in 6-well plates cultured for 7 days.

To investigate the role of miR-133b in GC carcinogenesis, we re-introduced miR-133b mimics into two GC cell lines, HGC-27 and MGC-803, in which miR-133b was down-regulated. miR-133b was successfully overexpressed in these two cell lines which was confirmed by q-PCR. As shown in Figure 2A and 2C, miR-133b was overexpressed about 16 folds and 18 folds than the scramble control or untreated cells in HGC-27 and MGC-803 cells respectively. Consistent with its low expression in GC, the overexpression of miR-133b in both of the two GC cell lines can inhibit GC cell proliferation significantly as demonstrated by CCK-8 growth assay. The scramble control had no effect on cell proliferation compared with the untreated cells (Figure 2B, D). We also detected the effect of miR-133b on the colony formation ability of GC cells. The mimic-transfected GC cells were replated at low density and maintained for 7 days. The overexpression of miR-133b significantly decreased the colony number of HGC-27 and MGC-803 cells, whereas the scramble control had little effect on the colony number compared with the untreated cells (Figure 2E, F). The results suggested that miR-133b might act as a tumor suppressor in GC.

### miR-133b can inhibit GC cell migration and invasion

**Figure 3:**
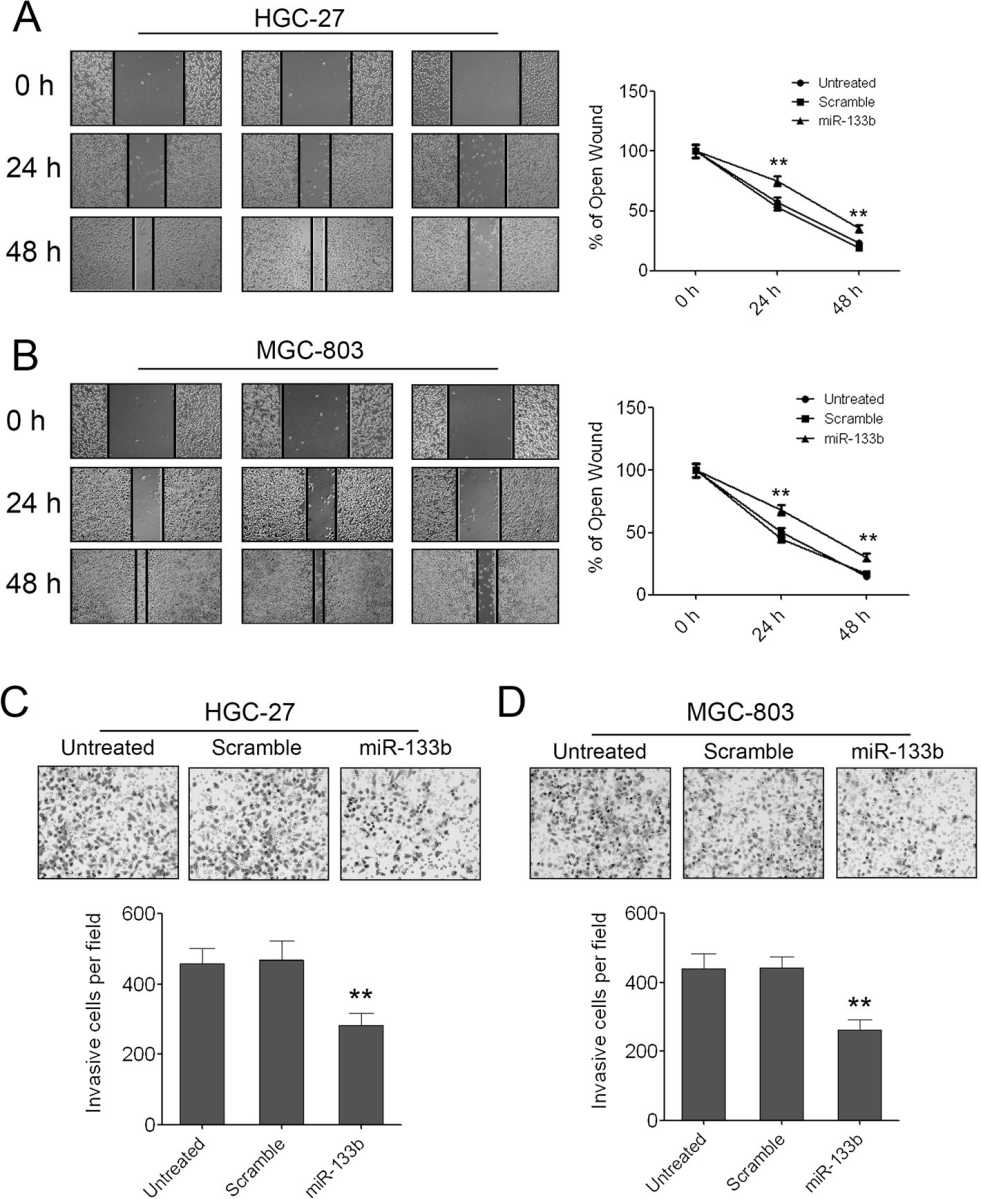
**Enforced expression of miR-133b can inhibit GC cell migration and invasion. (A)** The pictures of wound healing and the percentages of open wound of HGC-27 cells at 0, 24, 48 hours after scratching. Data are shown as mean + s.d. (n = 3); ** indicates P-value <0.01. **(B)** The pictures of wound healing and the percentages of open wound of MGC-803 cells at 0, 24, 48 hours after scratching. Data are shown as mean + s.d. (n = 3); ** indicates P-value <0.01. **(C)** The invaded HGC-27 cells in the Matrigel transwell invasion assay. Data are shown as mean + s.d. (n = 3); ** indicates P-value <0.01. **(D)** The invaded MGC-803 cells in the Matrigel transwell invasion assay. Data are shown as mean + s.d. (n = 3); ** indicates P-value <0.01.

We further assessed the effects of miR-133b on cell migration and invasion, which were the important determinants of malignant progression and metastasis. A wound healing/scratch assay was used to evaluate the migration ability of HGC-27 and MGC-803 cells. Both of the two cell lines treated with miR-133b mimic showed slow migration rate compared with the scramble control or untreated cells. The percentage of wound healing at 24 and 48 hours after scratching was significantly decreased in GC cells treated with miR-133b mimics (Figure 3A, B). Furthermore, we conducted a Matrigel cell invasion assay to assess the role of miR-133b in regulating cell invasion. The Matrigel cell invasion assay showed that overexpression of miR-133b in the two GC cell lines dramatically decreased the invaded cells compared with the scramble control and untreated cells (Figure 3C, D). These results indicated that miR-133b played important roles in regulating cell migration and invasiveness in gastric carcinogenesis.

### Knockdown of miR-133b in GES cells can promote cell proliferation and invasion

**Figure 4:**
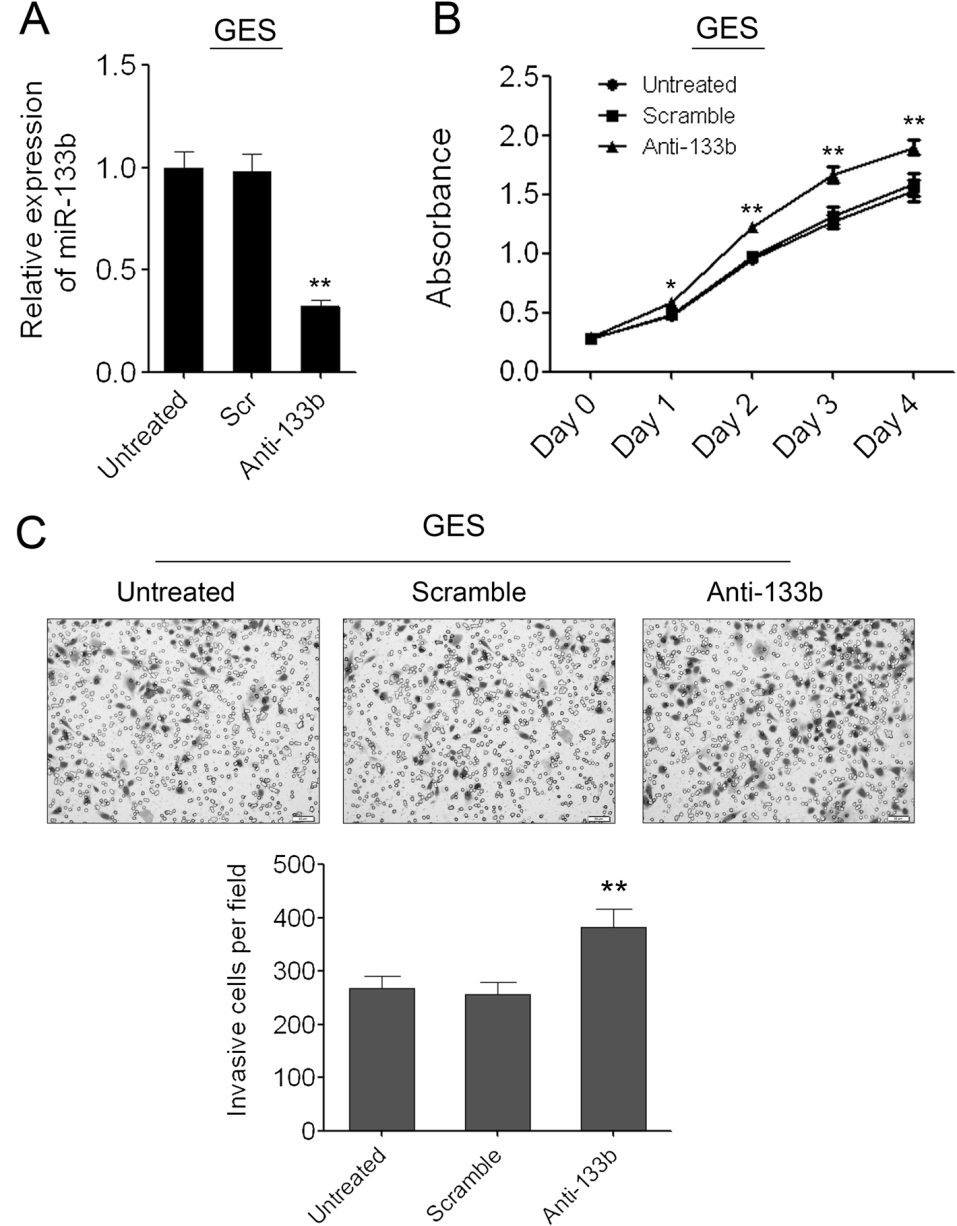
**Knockdown of miR-133b in GES cells can promote cell proliferation and migration. (A)** Inhibition of miR-133b in GES cells was confirmed by qRT-PCR. **(B)** The cell growth of GES cells at day 0, 1, 2, 3, 4 post transfection which was detected by CCK-8 assay. Data are shown as mean ± s.d. (n = 3); * indicates P-value <0.05;** indicates P-value <0.01. **(C)** The invaded GES cells in the Matrigel transwell invasion assay. Data are shown as mean + s.d. (n = 3); ** indicates P-value <0.01.

To further investigate the role of miR-133b in normal gastric epithelium cells, we introduced miR-133b inhibitors into GES cells. miR-133b was successfully reduced in GES cells which was confirmed by q-PCR (Figure 4A). Accompanied by the knockdown of miR-133b in GES cells, cell proliferation was promoted significantly as demonstrated by CCK-8 growth assay. The scramble control had no effect on cell proliferation compared with the untreated cells (Figure 4B). We also detected the effect of miR-133b on GES cell invasion. The Matrigel cell invasion assay showed that knockdown of miR-133b in GES cells dramatically increased the invaded cells compared with the scramble control and untreated cells (Figure 4C).

### miR-133b targets FSCN1 in GC cells

We further dissected the mechanism by which miR-133b functioned as a tumor suppressor in GC. miR-133b has been reported to directly target oncogenic FSCN1 gene in esophageal squamous cell carcinoma [26]. However, it was still unknown whether miR-133b played its tumor suppressor roles through targeting FSCN1 in GC.

**Figure 5:**
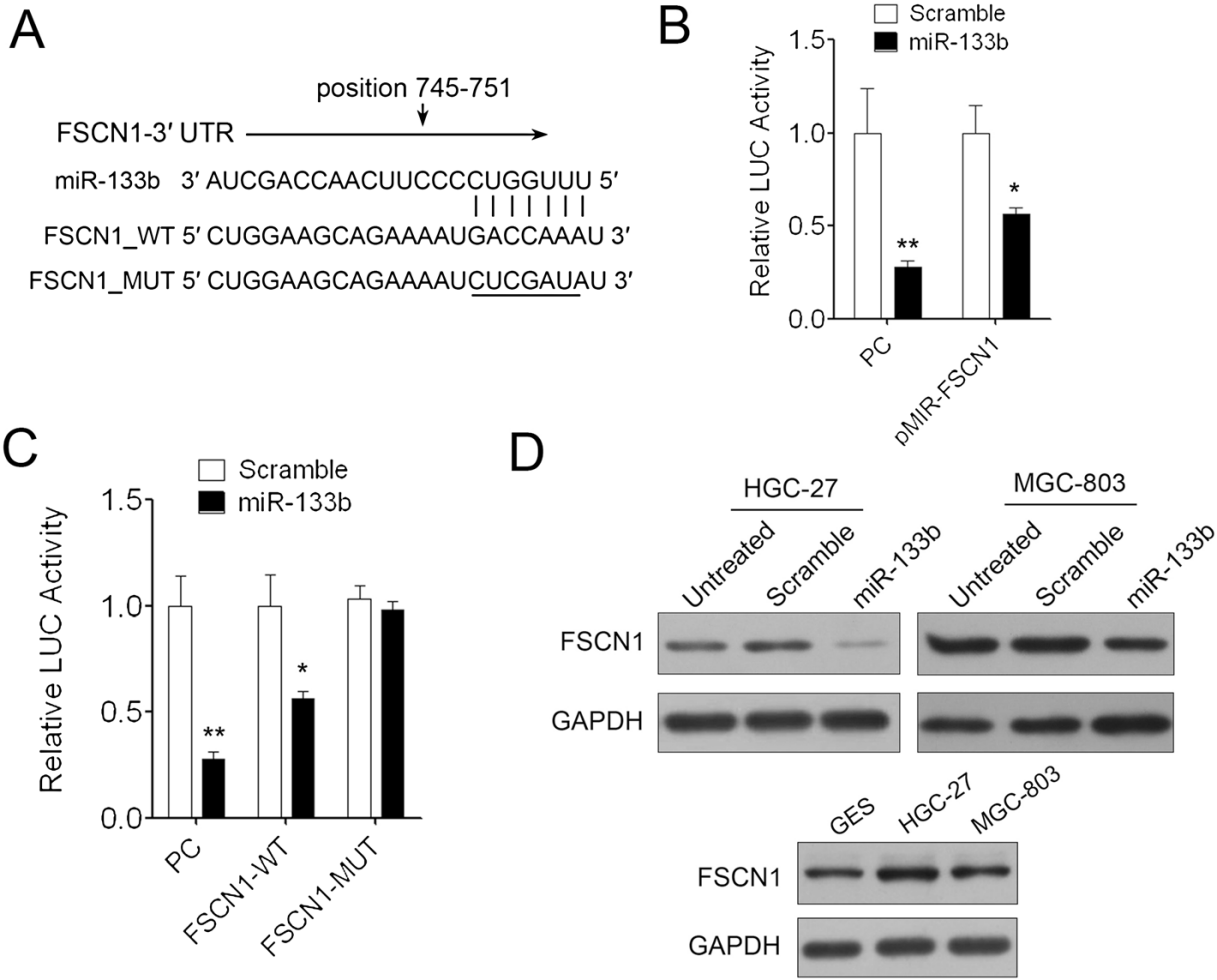
**FSCN1 is a direct target of miR-133b in GC cells. (A)** Schematic representation of FSCN1 3′ UTR showing putative miR-133b binding site. **(B)** Relative luciferase activity of FSCN1 3′ UTR reporter constructs in 293 T cells. Data are shown as mean + s.d. (n = 3); * indicates P-value <0.05, ** indicates P-value <0.01. **(C)** Relative luciferase activity of wild type and mutated FSCN1 3′ UTR constructs in 293 T cells. Data are shown as mean + s.d. (n = 3); * indicates P-value <0.05, ** indicates P-value <0.01. **(D)** Western blot analysis of FSCN1 expression in HGC-27 and MGC-803 cells transfected with scramble control or miR-133b mimics; FSCN1 expression level in GES, HGC-27 and MGC-803 cells.

miR-133b was predicted to bind to the 3′ -UTR region of FSCN1 using two algorithms PicTar(http://pictar.mdc-berlin.de/) and TargetScan(http://www.targetscan.org/) (Figure 5A). To validate the negative regulation of miR-133b on FSCN1, we cloned the 3′ -UTR of FSCN1 into a luciferase reporter construct (pMIR-reporter) meanwhile cloned nucleotide sequence complete complementary to miR-133b as positive control (PC). Dramatic decrease of luciferase activity of PC demonstrated that miR-133b was successfully expressed in 293 T cells and the reporter assay was convincing. The reporter assay also showed that miR-133b repressed the luciferase activity of FSCN1 3′ -UTR significantly compared with the scramble control (Figure 5B). To test whether the repression was dependent on the miRNA binding site, we constructed another luciferase reporter of FSCN1 3′ -UTR in which the miRNA binding site in the 3′ -UTR was mutated. The results indicated that mutation of the miRNA binding sites in the FSCN1 3′ -UTR abrogated the reduction in luciferase activities which suggested that the repression of miR-133b on FSCN1 3′ -UTR was dependent on the miRNA binding site (Figure 5C). Consistent with the reporter assay, we observed an evident decrease of FSCN1 protein in presence of miR-133b mimics compared to scramble control in both of HGC-27 and MGC-803 cells. Meanwhile, we also noticed that the expression level of FSCN1 in HGC-27 and MGC-803 cells was higher than that in GES cells which was consistent with low expression of miR-133b in GC cells (Figure 5D).

### Knock down of FSCN1 can inhibit GC cell growth and invasion

**Figure 6:**
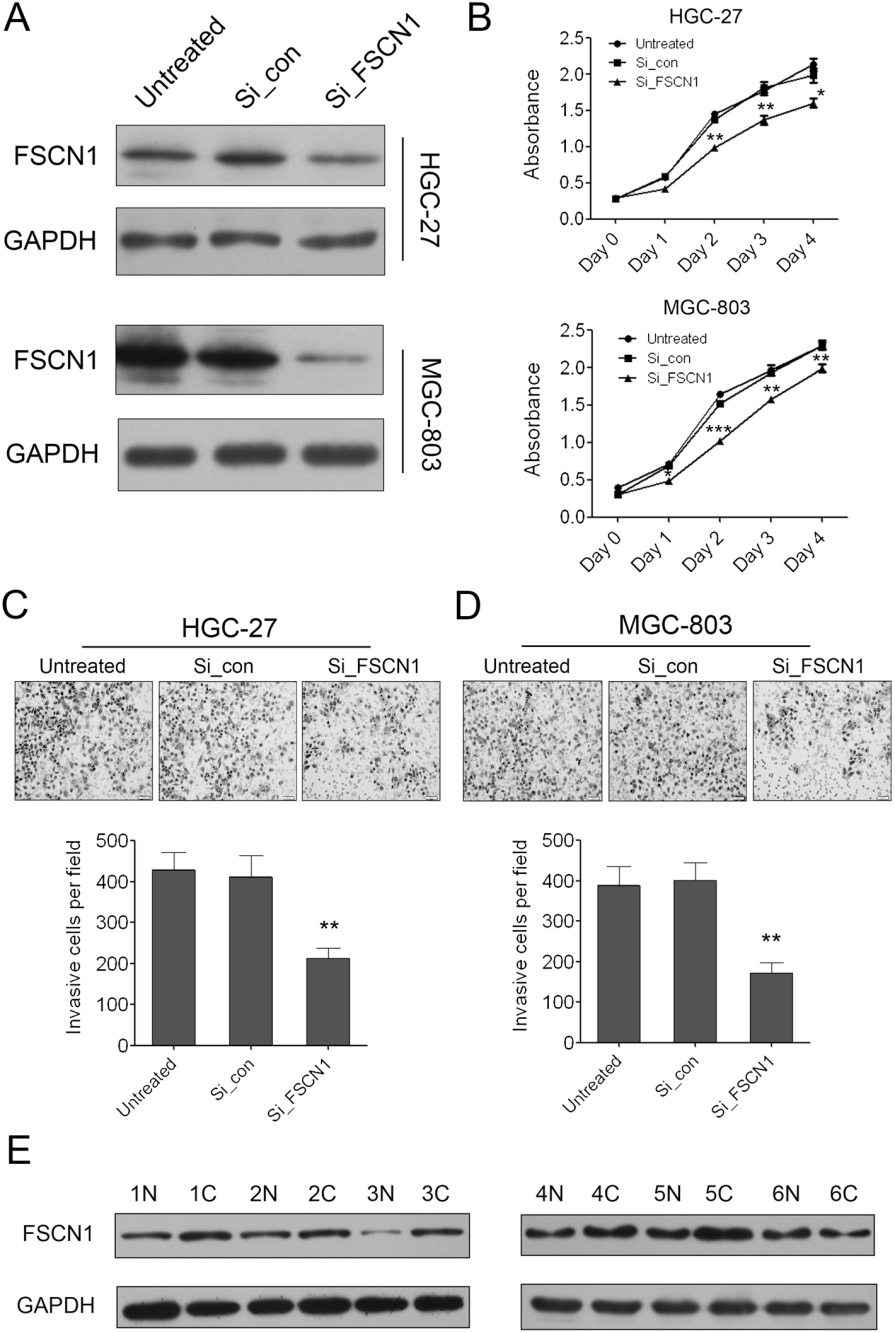
**Knock down of FSCN1 can inhibit GC cell growth and invasion. (A)** Western blot analysis of FSCN1 expression in HGC-27 and MGC-803 cells transfected with negative control or FSCN1 siRNAs. **(B)** The cell growth of HGC-27 and MGC-803 cells at day 0, 1, 2, 3, 4 post transfection which was detected by CCK-8 assay. Data are shown as mean + s.d. (n = 3); * indicates P-value <0.05. ** indicates P-value <0.01. *** indicates P-value <0.001. **(C)** The invaded HGC-27 cells in the Matrigel transwell invasion assay. Data are shown as mean + s.d. (n = 3); ** indicates P-value <0.01. **(D)** The invaded MGC-803 cells in the Matrigel transwell invasion assay. Data are shown as mean + s.d. (n = 3); ** indicates P-value <0.01. **(E)** Western blot analysis of FSCN1 expression in 6 pairs of GC tissues **(C)** and the adjacent non-neoplastic tissues (N).

To investigate the role of FSCN1 in GC carcinogenesis, we knocked down FSCN1 expression using siRNAs in two GC cell lines, HGC-27 and MGC-803. As shown in Figure 6A, the expression of FSCN1 was successfully knocked down in both of the two cell lines which was confirmed by Western blot. Knock down of FSCN1 attenuated GC cell proliferation significantly as demonstrated by CCK-8 growth assay, whereas negative control had no effect on cell proliferation compared with the untreated cells (Figure 6B). We also detected the effect of FSCN1 in regulating cell invasion using the Matrigel cell invasion assay. The results showed that repressed FSCN1 in the two GC cell lines dramatically decreased the invaded cells compared with the negative control and untreated cells (Figure 6C, D). Finally, we examined the protein level of FSCN1 in 6 randomly selected clinical samples in which miR-133b was down-regulated in their GC tissues. Among the total 6 GC patients we investigated, there were five cases in which FSCN1 was up-regulated along with the down-regulation of miR-133b in GC tissues indicating that miR-133b suppress the expression of FSCN1 in these patients (Figure 6E). These results indicated that FSCN1 might play important roles in GC as an oncogene and miR-133b regulated GC cell proliferation, migration and invasion through targeting FSCN1.

## Discussion

In this study, we investigated the expression of miR-133b in 100 patients with gastric cancer and dissected the roles and mechanisms of miR-133b in GC carcinogenesis. We found that miR-133b was significantly down-regulated in GC tissues and the low expression of miR-133b was associated with the more aggressive phenotypes of GC. Re-introduction of miR-133b into GC cells can obviously inhibit GC cell proliferation, migration and invasion and the tumor suppressor roles of miR-133b in GC was partially through targeting oncogenic FSCN1. These results suggested that miR-133b can be developed as a new diagnostic marker or therapeutic target for GC.

The aberrant expression of miRNAs in GC has been studied in recent years and a total of 139 differentially expressed miRNAs were reported in thirteen miRNA expression profiling studies that compared GC tissues with neighbouring noncancerous or normal gastric tissues [28]. miR-21, miR-18a, miR-17 and miR-20a were the most frequently reported to be up-regulated in GC tissues [29-36]. Whereas, miR-375 and miR-378 were often detected to be down-regulated in GC tissues [29-32,34]. Meanwhile, various miRNAs have been shown to function as either tumor suppressors (inhibiting oncogenic potential) or oncogenes (promoting oncogenic potential) in GC. For example, miR-106b-25 cluster was reported to play a key role in TGF-β1 mediated tumor suppressor pathway in GC and the upregulation of these miRNAs impairs the TGF-β1 pathway, interfering with the expression of p21 [37]. The oncogenic cluster miR-221-222 can suppress expression of cyclin-dependent kinase inhibitors (p21, p27, p57) in GC, thus promoting GC cell proliferation [38]. miR-148a also can regulate the cell cycle progression through targeting p27 and knock down of miR-148a can inhibit GC cell proliferation [39]. miRNA are also indicated to play important roles in multidrug resistance in GC. Because miRNAs are small, stable against degradation, easy to be detected and easy to deliver, these aberrant expression miRNAs in GC are attractive as potential biomarkers and new targets for gastric cancer therapy. However, the potential diagnostic and therapeutic roles of these miRNAs in more clinical samples are just at the beginning and need to be explored further.

Among the miRNAs which were aberrantly expressed in GC, miR-133b was firstly reported to be down-regulated in GC tissues in three patients by microRNA microarray assay [23]. Subsequently, the level of miR-133b was investigated in 19 cases of gastrointestinal stromal tumor (GIST) and miR-133b was reported to be downregulated in high-grade GISTs suggesting it might have an important role in the progression of GIST [24]. A recent study also identified miR-133b levels in 12 pairs of GC tissue samples and found that miR-133b expression was downregulated in 11/12 of the tested GC tissues compared with matched nontumor tissues [25]. In our study, we investigated the expression of miR-133b in large numbers of patients with gastric cancer firstly. The significant down-regulation of miR-133b in GC tissues was consistent with the previous report and also hinted that it can be developed as a potential biomarker for GC diagnosis. Furthermore, we also found that low-level expression of miR-133b was associated with aggressive phenotypes of gastric cancer. In addition, miR-133b has been reported to suppress GC cell proliferation through targeting FGFR1 [25]. We found that miR-133b not only can inhibit the GC cell proliferation but also could suppress the GC cell migration and invasion. To further investigate the tumor suppressor role of miR-133b and the detailed mechanism *in vivo* will be helpful for understanding the essential role of miR-133b in gastric cancer progression.

FSCN1 encodes a member of fascin family of actin-binding proteins. Fascin proteins organize F-actin into parallel bundles, and are required for the formation of actin-based cellular protrusions. FSCN1 plays important roles in cell migration, motility, adhesion and cellular interactions and act as an oncogene in multiple types of cancer by increasing cell motility [40]. Knockdown of fascin1 expression have been reported to be able to suppress the proliferation and metastasis of MKN45 gastric cancer cells [41]. Fascin-1 was also involved in Galectin-3, a beta-galactoside-binding protein, mediated gastric cancer cell motility increasement [42]. Moreover, higher expression of fascin-1 was also correlated directly with more-advanced cancer stages (TNM) and inversely with survival rates in gastric adenocarcinomas [43]. miR-133b has been reported to directly regulate FSCN1 in esophageal squamous cell carcinoma [26]. In our study, we indicated that FSCN1 was also a direct target of miR-133b in GC cells. We also demonstrated that knock down of FSCN1 can inhibit GC cell growth and invasion suggesting its oncogenic roles in GC. So we concluded that miR-133b suppressed GC cell proliferation, migration and invasion through targeting FSCN1. Moreover, the inverse correlation of FSCN1 and miR-133b has been improved in 19 cases of GIST. These studies suggested that the down-regulation of miR-133b and the resulting elevated FSCN1 level played critical role in GC carcinogenesis.

## Conclusion

In summary, we demonstrated the significant down-regulation of miR-133b in large numbers of GC patients and the lower expression of miR-133b in high grade GC patients. We also dissected the tumor suppressor roles of miR-133b in GC cells and found that it was partially through targeting oncogenic FSCN1.

## Consent

Written informed consent was obtained from the patient’s guardian/parent/next of kin for the publication of this report and any accompanying images.
