# Correction to: The role of microRNA-133b and its target gene FSCN1 in gastric cancer

**DOI:** 10.1186/s13046-020-01716-6

**Published:** 2020-10-20

**Authors:** Lihua Guo, Hua Bai, Dongling Zou, Tao Hong, Jie Liu, Jiaqiang Huang, Pengfei He, Qi Zhou, Jinsheng He

**Affiliations:** 1 School of Computer and Information Technology, Shangyuan Residence, Haidian District, Beijing, 100044 China; 2 College of Life Sciences and Bioengineering, Beijing Jiaotong University, Shangyuan Residence, Haidian District, Beijing, 100044 China; 3 Department of Ophthalmology, General Hospital of Bei Jing Command of PLA, #5 Nanmencang, DongCheng District, Beijing, 100700 China; 4 Department of Gynecologic Oncology, Chongqing Cancer Institute, Chongqing, 400030 China; 5 National Institutes for Food and Drug Control, No.2 Tiantan Xi Li, Beijing, 100050 China

**Correction to: J Exp Clin Cancer Res 33, 99 (2014)**

**https://doi.org/10.1186/s13046-014-0099-0**

Following publication of the article [1], the authors identified errors in Figs. 3, 4 and 6; specifically panels Fig. 3c (HGC-27 ‘untreated’), Fig. 4c (GES ‘untreated’), Fig. 6c and d. The corrections do not change the results or the conclusions of this paper.

**Fig. 3:**
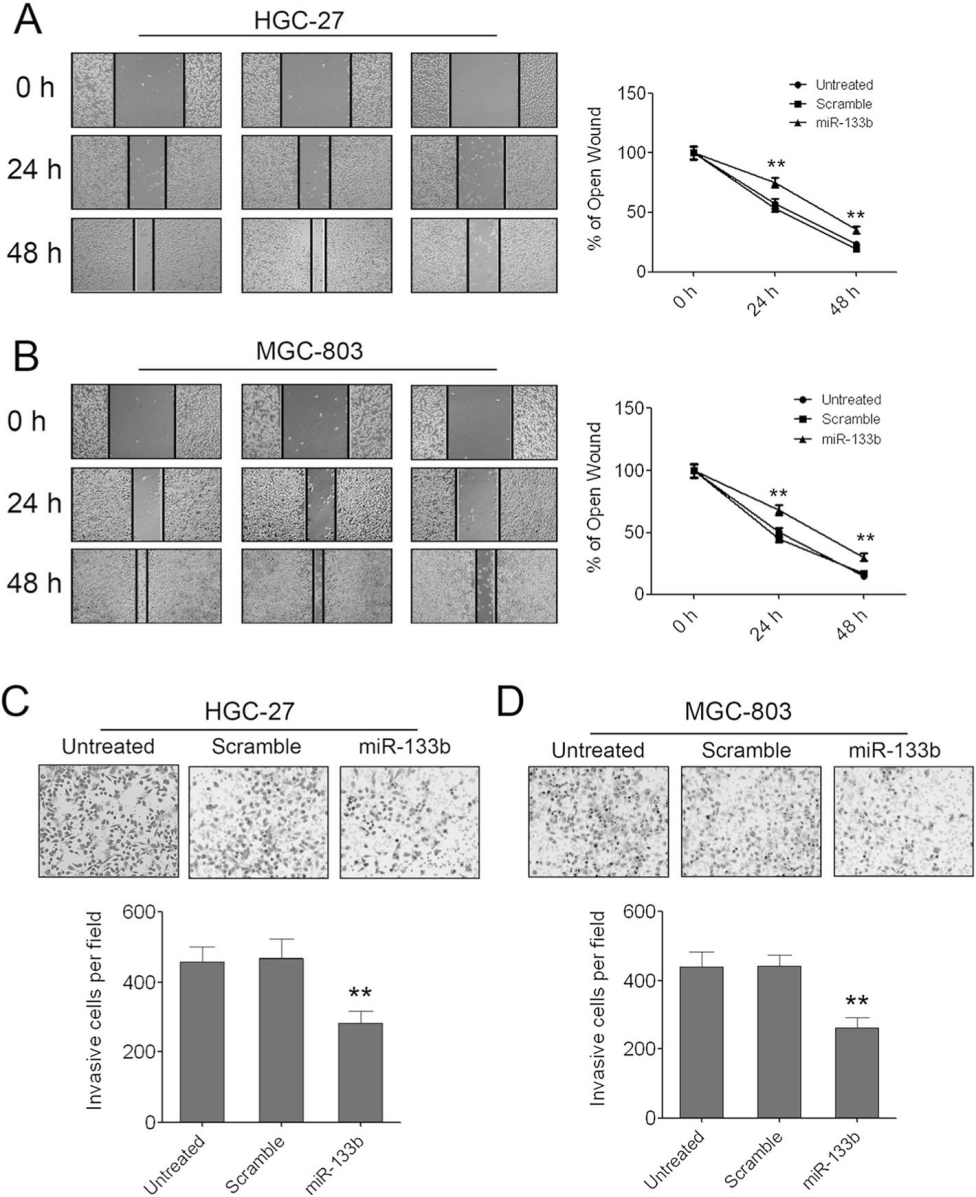
**Enforced expression of miR-133b can inhibit GC cell migration and invasion. (A)** The pictures of wound healing and the percentages of open wound of HGC-27 cells at 0, 24, 48 hours after scratching. Data are shown as mean + s.d. (n = 3); ** indicates P-value <0.01. **(B)** The pictures of wound healing and the percentages of open wound of MGC-803 cells at 0, 24, 48 hours after scratching. Data are shown as mean + s.d. (n = 3); ** indicates P-value <0.01. **(C)** The invaded HGC-27 cells in the Matrigel transwell invasion assay. Data are shown as mean + s.d. (n = 3); ** indicates P-value <0.01. **(D)** The invaded MGC-803 cells in the Matrigel transwell invasion assay. Data are shown as mean + s.d. (n = 3); ** indicates P-value <0.01

**Fig. 4:**
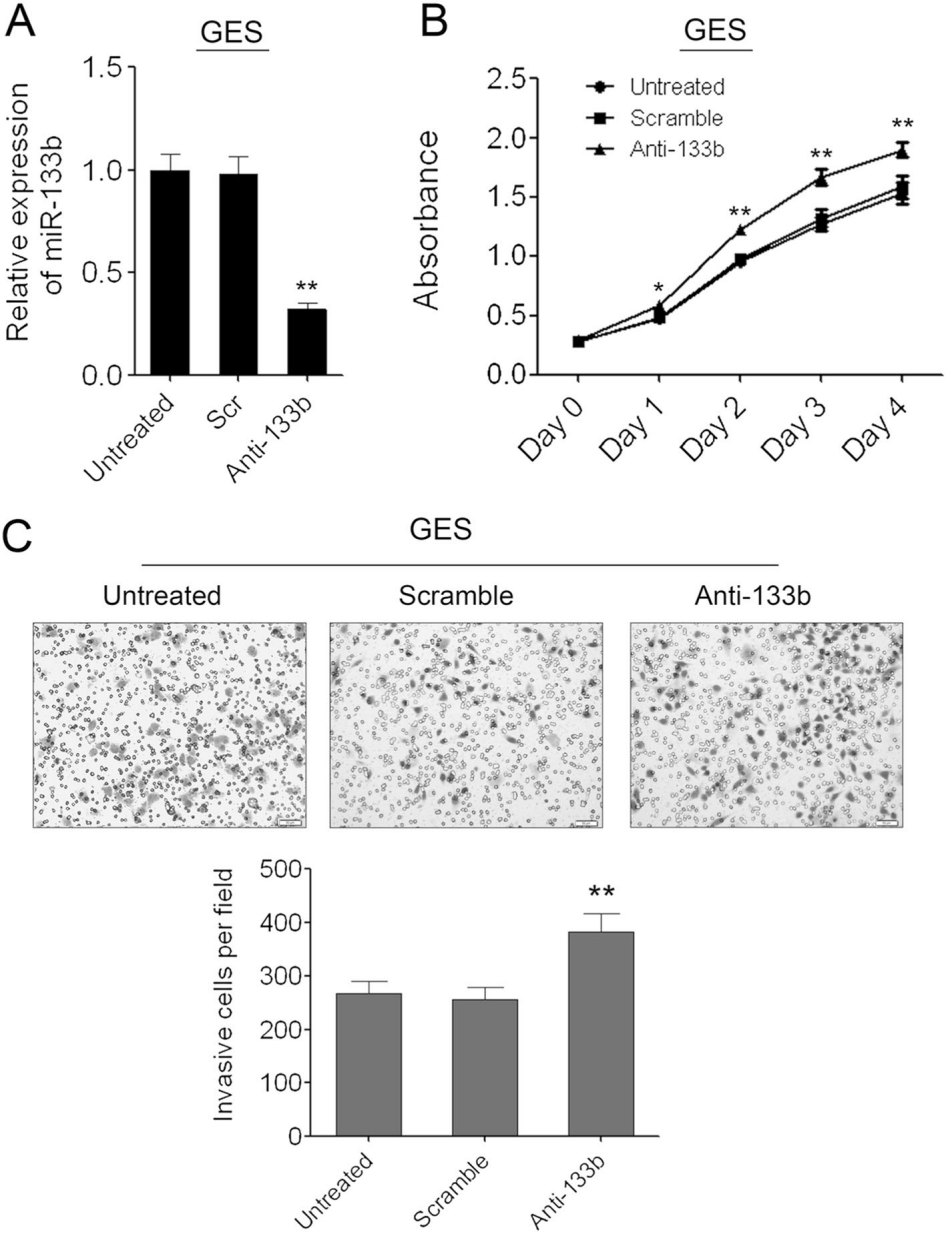
**Knockdown of miR-133b in GES cells can promote cell proliferation and migration. (A)** Inhibition of miR-133b in GES cells was confirmed by qRT-PCR. **(B)** The cell growth of GES cells at day 0, 1, 2, 3, 4 post transfection which was detected by CCK-8 assay. Data are shown as mean ± s.d. (n = 3); * indicates P-value <0.05;** indicates P-value <0.01. **(C)** The invaded GES cells in the Matrigel transwell invasion assay. Data are shown as mean + s.d. (n = 3); ** indicates P-value <0.01

**Fig. 6:**
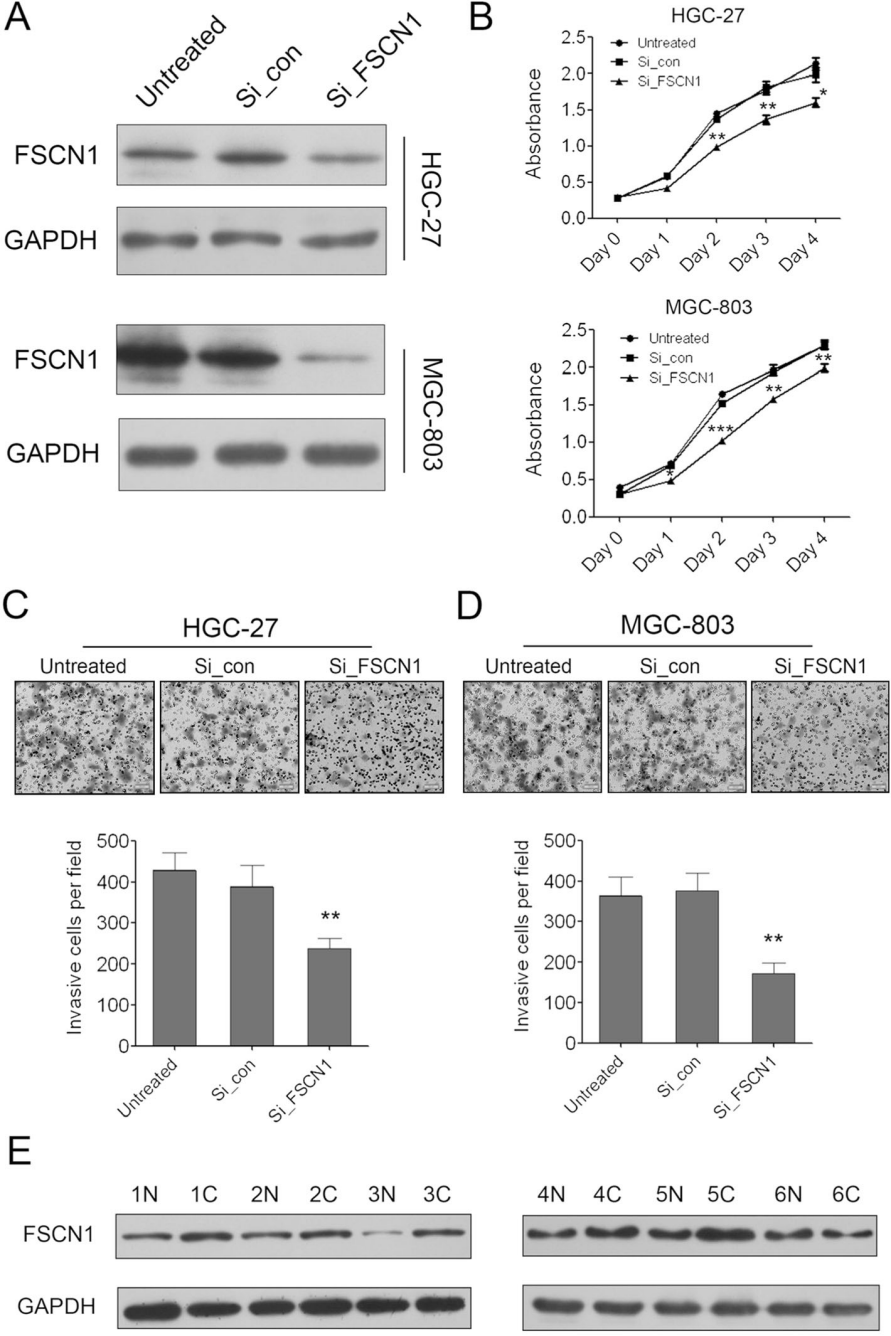
**Knock down of FSCN1 can inhibit GC cell growth and invasion. (A)** Western blot analysis of FSCN1 expression in HGC-27 and MGC-803 cells transfected with negative control or FSCN1 siRNAs. **(B)** The cell growth of HGC-27 and MGC-803 cells at day 0, 1, 2, 3, 4 post transfection which was detected by CCK-8 assay. Data are shown as mean + s.d. (n = 3); * indicates P-value <0.05. ** indicates P-value <0.01. *** indicates P-value <0.001. **(C)** The invaded HGC-27 cells in the Matrigel transwell invasion assay. Data are shown as mean + s.d. (n = 3); ** indicates P-value <0.01. **(D)** The invaded MGC-803 cells in the Matrigel transwell invasion assay. Data are shown as mean + s.d. (n = 3); ** indicates P-value <0.01. **(E)** Western blot analysis of FSCN1 expression in 6 pairs of GC tissues **(C)** and the adjacent non-neoplastic tissues (N)
